# Around the Hospitals

**Published:** 1897-08-21

**Authors:** 


### AROUND THE HOSPITALS.

[Notice to the Managers of Institutions.—Special space is now reserved for the insertion of notices of hospital meetings and festivals. The Editor requests that all notices may reach The Hospital Office, 28 & 29, Southampton Street, Strand, London, W.C., at least one week before the date of each meeting.]

**North-Eastern Hospital for Children.**—In consequence of the demolition, owing to decay, of one of the houses forming part of the freehold property of this hospital, which provided accommodation for ten members of the household, the authorities of the North-Eastern Hospital for Children are now being obliged to hasten to a finish the scheme of providing a hospital which shall supply at least 100 beds for the district it serves. This scheme, which was proposed in 1875, has never been fully carried out owing to the straitened circumstances under which the general funds of the hospital have continually suffered, only the out-patient department and wards providing accommodation for 57 in-patients having been opened, the remaining ward having been left unbuilt. Matters have now been brought to a head by the urgency of the need for more room in every department of the hospital, and an appeal is being made for £20,000 to build the completing wing, of which sum the committee have in hand £5,000, the half of a legacy left to the hospital in 1893 by the late Mr. John Horniman. That the need is urgent is shown by the fact that within one mile radius of the hospital there is a population of over 360,000, of whom nearly one-half, or about 170,000, are children under 12 years of age, the vast majority of whom belong to the poorer classes. For the needs of these children who live, as can well be believed, under not too healthy conditions, there are only 94 beds provided, viz., 57 at the North-Eastern, 20 at the Mildmay Mission Hospital, and 17 at the Metropolitan Hospital, or only one bed for every 1,800 children. In consequence of this, the pressure on the beds already provided by the North-Eastern Hospital is so great that a considerable proportion of the out-patients are cases which really require in-patient treatment, but for whom no room can be found in the wards. Besides this, the present building is very deficient in accommodation for the nurses and the household; the ward originally provided for isolation is constantly filled with severe cases of diphtheria; there is no proper mortuary; and other necessary offices are non-existent. Taking all these circumstances into consideration, the hospital authorities appear to have made out an excellent case for presentation to the public.

**Royal Hants County Hospital.**—The question of instituting a new system of drainage at this hospital has been under consideration for some time past, and plans have been prepared which include, in addition, new lavatories in place of those now opening into the ward kitchens, a receiving-room for patients before being passed into the wards, a new office for the secretary, and a patients' entrance to the hospital. Tenders for the work have been invited. The scheme was submitted to Sir Douglas Galton, who has always taken an especial interest in the hospital, and who was consulted regarding the plans of the existing building when it was erected some thirty years ago, and he has expressed his full approval of it. When this scheme is carried out the authorities will have to turn their attention to the provision of wards for children, who this year have been removed from the adult wards and placed in one of the special wards; and of further provision for the nurses, another of the special wards having to be utilised as a sleeping-room owing to the present inadequate accommodation. During the year ended March 31st, 1897, 705 in and 727 out-patients were treated, the corresponding figures for the previous year being 619 and 666. Sixty-eight out of the 108 beds provided were on an average occupied by the patients daily, and each patient remained on an average 46 days. The accounts, which are made up on the uniform system, show the ordinary receipts to have been £5,080, and legacies £450; while the ordinary expenditure was £5,327, and the extraordinary expenditure £131. Subscriptions in arrear seem to be of rather a large amount, seeing that out of the £1,727 received in 1896-7 no less than £450, or, roughly speaking, one-fourth, were payments of arrears. Another £200 of arrears had even then not been paid. The convalescent home at Bonchurch in connection with this hospital received 91 patients during the year; but why is it that only 22, or less than a quarter of them, were hospital patients, and the remainder private patients, when the rules lay down that "the home is intended primarily for the reception of convalescent patients from the Royal Hants County Hospital?"

**Perth Royal Infirmary.**—The ordinary income of the County and City of Perth Royal Infirmary for the year 1896 only amounted to £2,527, while the ordinary expenditure amounted to £3,749, leaving a deficiency of £1,222 to be met out of the £4,365 received from legacies during the year. This expenditure is £242 higher than that for 1895, owing principally to the increase of 40 in the number of in-patients treated. 803 in-patients were under treatment in 1896, 706 were treated at the dispensary, 234 were visited at their homes by the district surgeon, and 1,094 casualties attended. On an average each in-patient stayed for 26 days, as against 25·7 days in 1895, while the daily average number was 61, against 54 in the previous year. Under agreement with the local authorities a large number of infectious cases are admitted—155 in 1896 and 210 in 1895—which adds considerably to the cost per head.

